# A numerical solution by alternative Legendre polynomials on a model for novel coronavirus (COVID-19)

**DOI:** 10.1186/s13662-020-02984-4

**Published:** 2020-09-25

**Authors:** Elham Hashemizadeh, Mohammad Ali Ebadi

**Affiliations:** 1 Department of Mathematics, Karaj Branch, Islamic Azad University, Karaj, Iran; 2 Young Researchers and Elite Club, Karaj Branch, Islamic Azad University, Karaj, Iran

**Keywords:** Coronavirus, COVID-19, Operational matrix of derivative, Alternative Legendre polynomials

## Abstract

Coronavirus disease (COVID-19) is an infectious disease caused by a newly discovered coronavirus. This paper provides a numerical solution for the mathematical model of the novel coronavirus by the application of alternative Legendre polynomials to find the transmissibility of COVID-19. The mathematical model of the present problem is a system of differential equations. The goal is to convert this system to an algebraic system by use of the useful property of alternative Legendre polynomials and collocation method that can be solved easily. We compare the results of this method with those of the Runge–Kutta method to show the efficiency of the proposed method.

## Introduction

An outbreak of the 2019 novel coronavirus disease (COVID-19) in Wuhan, China has spread quickly nationwide. The COVID-19 epidemic has spread very quickly from China to all the world [1, 2]. Countries continue to battle the novel coronavirus as it has infected more than 28 million around the world [3].

In [4] the COVID-19 mathematical model has been derived as follows, where $S_{p} ( t )$ is susceptible people, $E_{p} ( t )$ is exposed people, $I_{p} ( t )$ is symptomatic infected people, $A_{p} ( t )$ is asymptomatic infected people, $R_{p} ( t )$ is recovered and dead people, and $W ( t )$ is COVID-19 in reservoir in time *t*. The parameters needed are defined in Table 1 and $\Lambda _{p} = n_{p} \times N_{p}$, where $N_{p}$ refers to the total number of people:

1 $$ \textstyle\begin{cases} \frac{dS_{p}}{dt} = \Lambda _{p} - m_{p}S_{p} - \beta _{p}S_{p} ( I_{p} + kA_{p} ) - \beta _{W}S_{p}{W}, \\ \frac{dE_{p}}{dt} = \beta _{p}S_{p} ( I_{p} + kA_{p} ) + \beta _{W}S_{p}W - ( 1 - \delta _{p} )\omega _{p}E_{p} - \delta _{p}\omega '_{p}E_{p} - m_{p}E_{p}, \\ \frac{dI_{p}}{dt} = ( 1 - \delta _{p} )\omega _{p}E_{p} - ( \gamma _{p} + m_{p} )I_{p}, \\ \frac{dA_{p}}{dt} = \delta _{p}\omega '_{p}E_{p} - ( \gamma '_{p} + m_{p} )A_{p}, \\ \frac{dR_{p}}{dt} = \gamma _{p}I_{p} + \gamma '_{p}A_{p} - m_{p}R_{p}, \\ \frac{dW}{dt} = \mu _{p}{I}_{p} + \mu '_{p}{A}_{p} - \varepsilon {W}. \end{cases} $$

This paper aims to find the transmissibility of the COVID-19 by finding the unknowns $S_{p}$, $E_{p}$, $I_{p}$, $A_{p}$, $R_{p}$, and *W*. In medical sciences, the computation of these variables is vital to measure the progression of disease and to get a better cure.

**Table 1:** Definition of the parameters in the COVID-19 model

Variables and parameters	Descriptions
$n_{p}$	The birth rate of people
$m_{p}$	The death rate of people
$\frac{1}{\omega _{p}}$	The incubation period of people
$\frac{1}{\omega '_{p}}$	The latent period of people
$\frac{1}{\gamma _{p}}$	The infectious period of symptomatic infection in people
$\frac{1}{\gamma '_{p}}$	The infectious period of asymptomatic infection in people
$\mu _{p}$	The shedding coefficients from $I_{P}$ to *W*
$\mu '_{p}$	The shedding coefficients from $A_{p}$ to *W*
$\delta _{p}$	The proportion of asymptomatic infection rate of people
$\beta _{p}$	The transmission rate from $I_{p}$ to $S_{p}$
$\beta _{W}$	The transmission rate from *W* to $S_{p}$
*k*	The multiple of the transmissibility of $A_{p}$ to that of $I_{p}$
$\frac{1}{\varepsilon } $	The lifetime of the virus in *W*
*c*	The relative shedding rate of $A_{p}$ compared to $I_{p}$

In this paper, for finding these variables, we use alternative Legendre polynomials and their operational matrix of derivative. The proposed method results are compared to those of Runge–Kutta method, which shows the reliability of the proposed method.

There exist some related papers on this topic that have solved the coronavirus model or some differential equation system that appears in the disease model, so we refer the readers to them to see some similar methods on this topic [5–8].

The remainder of the article is organized as follows. In Sect. 2, we review the properties of alternative Legendre polynomials and approximation of a function with them. Then we present the operational matrix of derivatives of these polynomials. In Sect. 3, we implement the alternative Legendre polynomials method on the coronavirus model. Section 4 shows the applicability of the proposed method through a test problem, also the results are compared with Runge–Kutta method results that confirm the reliability of the proposed method. Then Sect. 5 concludes the paper.

## Some basic concepts of alternative Legendre polynomials (ALPs)

### Properties of ALPs

The set ${P}_{n} = \{ P_{nk}:k = 0,1,\ldots,n\}$ of alternative Legendre polynomials of degree *n* is defined by an explicit formula on the interval $[0,1]$ (see [9]) as follows:

2 $$ P_{nk}(t) = \sum_{j = 0}^{n - k} ( - 1)^{j} \begin{pmatrix} n - k \\ j \end{pmatrix} \begin{pmatrix} n + k + j + 1 \\ n - k \end{pmatrix}t^{k + j},\quad k = 0,1,\ldots,n. $$

They are orthogonal on the interval $[0,1]$ with the weight function $w(t) = 1$. The ALPs satisfy the orthogonality relationships

3 $$ \int _{0}^{1} P_{nk}(t)P_{nl}(t) \,dt = \textstyle\begin{cases} \frac{1}{k + l + 1},& k = l, \\ 0,& k \ne l, \end{cases}\displaystyle \quad k,l = 0,1,\ldots,n. $$

We can reproduce Eq. (2) with Rodrigues’s type as follows:

4 $$ \begin{gathered} P_{nk}(t) = \frac{1}{(n - k)!} \frac{1}{t^{k + 1}}\frac{d^{n - k}}{dt^{n - k}} \bigl( t^{n + k + 1}(1 - t)^{n - k} \bigr), \\ \quad k = 0,1,\ldots,n. \end{gathered} $$

So, we have

5 $$ \int _{0}^{1} P_{nk}(t) \,dt = \int _{0}^{1} t^{n}\,dt = \frac{1}{n + 1},\quad k = 0,1,\ldots,n. $$

Here, we note that each element of the set ${P}_{n} = \{ P_{nk}\}_{k = 0}^{n}$ is the polynomial of other *n*. For example, in the following we introduce the alternative Legendre polynomials ${P}_{3} = \{ P_{nk}\}_{k = 0}^{3}$ ($n = 3$).

$$ \begin{gathered} P_{30}(t) = 4 - 30t + 60t^{2} - 35t^{3},\qquad P_{31}(t) = 10t - 30t^{2} + 21t^{3},\\ P_{32}(t) = 6t^{2} - 7t^{3},\qquad P_{33}(t) = t^{3}. \end{gathered} $$

**Figure 1:**
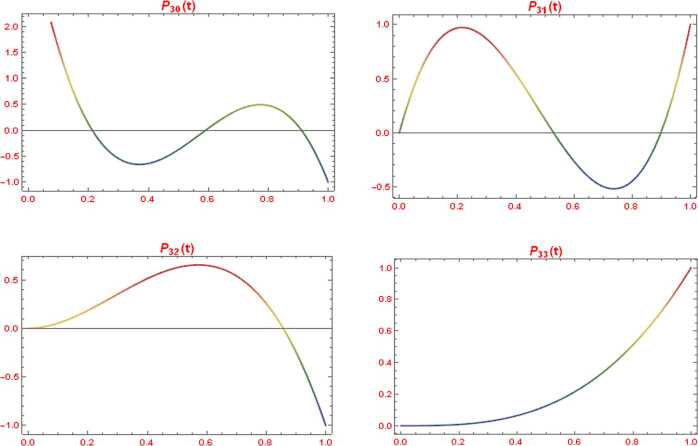
Plot of the ALPs with $n = 3$ on the interval $[0,1]$

In Fig. 1, we display the 4 set of ALPs with $n = 3$ over the interval $[0,1]$.

### Function approximation

Consider ${P}_{n} = \{ P_{nk}\}_{k = 0}^{n} \subset H = L^{2} [ 0,1 ]$ to be a set of ALPs and suppose that $Y = \operatorname{Span} \{ P_{nk} ( t ):k = 0,1, \ldots ,n \} $. So, *Y* is a finite dimensional subspace of *H*. Suppose, *f* to be an arbitrary function in *H*. Therefore, based on the Weierstrass theorem, every continuous function $f(t)$ on the interval [$a,b$] can be uniformly approximated by a polynomial function [9]. So, *f* has a unique best approximation in *Y* that we call $f^{*}(t)$. We have

6 $$ \bigl\Vert f ( t ) - f^{*} ( t ) \bigr\Vert _{2} \le \bigl\Vert f ( t ) - y ( t ) \bigr\Vert _{2}:\quad \forall y ( t ) \in Y. $$

Then this implies that

7 $$ \bigl\langle y,f - f^{*} \bigr\rangle = 0:\quad \forall y(t) \in Y, $$

where $\langle \cdot,\cdot \rangle $ denotes an inner product. Therefore, any arbitrary function $f \in H = L^{2} [ 0,1 ]$ may be approximated in terms of ALPs. So, there exists a set of unique coefficients $\{ c_{k}:k = 0,1, \ldots ,n \} $ such that

8 $$ f(t) \approx f^{*}(t) = \sum_{k = 0}^{n} c_{k}P_{nk}(t), $$

coefficient $c_{k}$ can be obtained in the following form:

9 $$ c_{k} = \frac{ \langle f,P_{nk} \rangle }{ \langle P_{nk},P_{nk} \rangle } = (2k + 1) \langle f,P_{nk} \rangle ,\quad k = 0,1,\ldots,n, $$

and

10 $$ \langle f,f \rangle = \int _{0}^{1} f^{2}(t)\,dt. $$

Also, Eq. (8) can be written in a matrix form as follows:

11 $$ f(t) \simeq \sum_{k = 0}^{n} c_{k}P_{nk}(t) = C^{T}\Phi (t), $$

where

12 $$ C = [c_{0},c_{1},\ldots,c_{n}] $$

and

13 $$ \Phi (t) = \bigl[P_{n0}(t),P_{n1}(t),\ldots,P_{nn}(t) \bigr]^{T}. $$

Let $a_{kj}^{(n)} = ( - 1)^{j}\binom {n - k }{j}\binom {n + k + j + 1}{n - k} $, then Eq. (2) can be written as

14 $$ P_{nk}(t) = \sum_{j = 0}^{n - k} a_{kj}^{(n)}t^{k + j},\quad k = 0,1, \ldots,n. $$

By using Eq. (14), for $k = 0,1,\ldots,n$ now, we can write

15 $$ \begin{aligned} \Phi (t) &= \begin{bmatrix} P_{n0}(t) \\ P_{n1}(t) \\ \vdots \\ P_{nn}(t) \end{bmatrix} = \begin{bmatrix} \sum_{j = 0}^{n} a_{0j}^{(n)}t^{j} \\ \sum_{j = 0}^{n - 1} a_{1j}^{(n)}t^{j + 1} \\ \vdots \\ \sum_{j = 0}^{n - n} a_{nj}^{(n)}t^{j + n} \end{bmatrix} = \begin{bmatrix} a_{00}^{(n)} + a_{01}^{(n)}t + a_{02}^{(n)}t^{2} + \cdots + a_{0n}^{(n)}t^{n} \\ a_{01}^{(n)}t + a_{02}^{(n)}t^{2} + \cdots + a_{0(n - 1)}^{(n)}t^{n} \\ \vdots \\ 0 \end{bmatrix} \\ &= \begin{bmatrix} a_{00}^{(n)} & a_{01}^{(n)} & \cdots & a_{0n}^{(n)} \\ 0 & a_{01}^{(n)} & \cdots & a_{0(n - 1)}^{(n)} \\ \vdots & \vdots & \ddots & \vdots \\ 0 & 0 & \cdots & a_{nn}^{(n)} \end{bmatrix} \begin{bmatrix} 1 \\ t \\ \vdots \\ t^{n} \end{bmatrix} = \Phi X_{t}. \end{aligned} $$

Therefore Eq. (13) can be written in the following form:

16 $$ \Phi (t) = \Phi X_{t}, $$

where

17 $$ X_{t} = \bigl[1,t,t^{2},\ldots,t^{n} \bigr]^{T}, $$

and Φ is the upper triangular matrix defined by [10]

18 $$ \begin{gathered} \Phi = [q_{kj}],\quad k,j = 0,1,\ldots,n, \\ q_{kj} = \textstyle\begin{cases} 0,& 0 \le j < k, \\ ( - 1)^{j - k}\binom {n - k}{j - k} \binom {n + j + 1}{n - k} ,& k \le j \le n. \end{cases}\displaystyle \end{gathered} $$

#### Definition

The tensor product of two vectors $f_{\hat{m}} = [ f_{i} ]$ and $g_{\hat{m}} = [ g_{i} ]$ is defined as

19 $$ f \otimes g = ( f_{i} \times g_{i} )_{\hat{m}}. $$

Similarly, for two matrices $A = [ a_{i,j} ]$ and $B = [ b_{i,j} ]$ of $\hat{m} \times \hat{m}$,

20 $$ A \otimes B = ( a_{i,j} \times b_{i,j} )_{\hat{m} \times \hat{m}}. $$

The lemma below will be needed in Sect. 3.

#### Lemma

*Let the functions* $f ( t )$, $g ( t ) \in L^{2} [ 0,1 ]$*be expanded into ALPs*, *that is*, $f ( t ) = f^{T}\Phi ( t )$*and* $g ( t ) = g^{T}\Phi ( t )$. *Then*

21 $$ f ( t )g ( t ) = \bigl( f^{T} \otimes g^{T} \bigr)\Phi ( t ). $$

#### Proof

$$ f ( t )g ( t ) = f^{T}\Phi ( t )\Phi ^{T} ( t )g = f_{1}g_{1}p_{n0} ( t ) + f_{2}g_{2}p_{n1} ( t ) + \cdots + f_{n}g_{n}p_{nn} ( t ) = \bigl( f^{T} \otimes g^{T} \bigr)\Phi ( t ). $$

 □

### Operational matrix of derivative

In this section, we derive the operational matrix of derivative of the ALPs that plays an important role in simplifying a system of differential equations and implementation of the proposed method.

To compute this operational matrix, we need to introduce the following properties of ALPs that can easily be deduced from the given definitions. Let $P_{ni}(t) = \sum_{r = 0}^{n} p_{r}^{(i)}t^{r}$, $P_{nj}(t) = \sum_{r = 0}^{n} p_{r}^{(j)}t^{r}$, and $P_{nk}(t) = \sum_{r = 0}^{n} p_{r}^{(k)}t^{r}$ be *i*th, *j*th, and *k*th of ALPs, respectively. Therefore, we have

22 $$\begin{aligned}& \bullet \quad \int _{0}^{1} t^{r}P_{nk}(t)\,dt = \sum_{l = 0}^{n - k} \frac{( - 1)^{l}\binom {n - k }{l} \binom {n + k + l + 1}{n - k} }{k + l + r + 1},\quad k = 0,1,\ldots,n. \end{aligned}$$

23 $$\begin{aligned}& \bullet \quad P_{nk}(t)P_{nj}(t) = \sum _{r = 0}^{2n} q_{r}^{(k,j)}t^{r}, \qquad q_{r}^{(k,j)} = \textstyle\begin{cases} \sum_{l = 0}^{r} p_{l}^{(k)}p_{r - l}^{(j)}, &r \le n, \\ \sum_{l = r - n}^{n} p_{l}^{(k)}p_{r - l}^{(j)}, &r > n, \end{cases}\displaystyle \end{aligned}$$

24 $$\begin{aligned}& \bullet \quad \int _{0}^{1} P_{ni}(t)P_{nj}(t)P_{nk}(t)\,dt = \sum_{r = 0}^{2n} q_{r}^{(k,j)} \sum_{l = 0}^{n - i} \frac{( - 1)^{l}\binom {n - i }{l} \binom {n + i + l + 1 }{n - i} }{i + l + r + 1} . \end{aligned}$$

The derivative of the vector $\Phi (t)$ can be expressed by

25 $$ \frac{d\Phi (t)}{dt} = D^{(1)}\Phi (t). $$

Here, $D^{(1)}$ is the $(n + 1) \times (n + 1)$ operational matrix of derivative.

So, by applying the differential operator with respect to *t*, we can write $D_{t} = \frac{d}{dt}$ (see[11]). By applying the polynomial $P_{nk}(t)$, we obtain

26 $$ \begin{aligned} D_{t}p_{nk}(t) &= D_{t}\sum _{j = 0}^{n - k} ( - 1)^{j} \begin{pmatrix} n - k \\ j \end{pmatrix} \begin{pmatrix} n + k + j + 1 \\ n - k \end{pmatrix}t^{k + j} \\ &= \sum_{j = 0}^{n - k} ( - 1)^{j} \begin{pmatrix} n - k \\ j \end{pmatrix} \begin{pmatrix} n + k + j + 1 \\ n - k \end{pmatrix}D_{t}t^{k + j} \\ &= \sum_{j = 0}^{n - k} ( - 1)^{j}(k + j) \begin{pmatrix} n - k \\ j \end{pmatrix} \begin{pmatrix} n + k + j + 1 \\ n - k \end{pmatrix}t^{k + j - 1}, \\ &\quad k = 0,1,\ldots,n. \end{aligned} $$

Here, by using Eq. (11), one can approximate $t^{k + j - 1}$ in terms of ALPs as follows:

27 $$ t^{k + j - 1} \simeq \sum_{r = 0}^{n} b_{r}^{(k,j)}p_{nr}(t). $$

The approximation coefficients $b_{r}^{(k,j)}$ are obtained using Eq. (9) as follows:

28 $$ \begin{aligned} b_{r}^{(k,j)} &= (2r + 1) \int _{0}^{1} y(t)P_{nr}\,dt \\ &= ( 2r + 1 ) \int _{0}^{1} t^{k + j - 1}\sum _{l = 0}^{n - r} ( - 1)^{l} \begin{pmatrix} n - r \\ l \end{pmatrix} \begin{pmatrix} n + r + l + 1 \\ n - r \end{pmatrix}t^{r + l} \,dt \\ &= ( 2r + 1 )\sum_{l = 0}^{n - r} \frac{( - 1)^{l}\binom {n - r }{l} \binom {n + r + l + 1 }{n - r} }{k + j + r + l},\quad r = 0,1,\ldots,n. \end{aligned} $$

Substituting (27) into (26), we have

29 $$ \begin{gathered} D_{t}p_{nk}(t) = \sum _{j = 0}^{n - k} ( - 1)^{j} \begin{pmatrix} n - k \\ j \end{pmatrix} \begin{pmatrix} n + k + j + 1 \\ n - k \end{pmatrix}\frac{\Gamma (k + j + 1)}{\Gamma (k + j + \alpha + 1)}\sum_{r = 0}^{n} b_{r}^{(k,j)}p_{n r}(t), \\ \quad k = 0,1,\ldots,n. \end{gathered} $$

Then, using Eqs. (26) and (27), we have

30 $$ \begin{aligned} &D_{t}p_{nk}(t) \\ &\quad = \sum _{r = 0}^{n} ( 2r + 1 )\\ &\qquad {}\times \left [ \sum _{j = 0}^{n - k} ( - 1)^{j}(k + j) \begin{pmatrix} n - k \\ j \end{pmatrix} \begin{pmatrix} n + k + j + 1 \\ n - k \end{pmatrix}\sum_{l = 0}^{n - r} \frac{( - 1)^{l}\binom {n - r }{l} \binom {n + r + l + 1 }{n - r} }{k + j + r + l} \right ] p_{n r}(t) \\ &\quad = \sum_{r = 0}^{n} \theta _{kr}^{(1)} p_{nr}(t) \end{aligned} $$

hence

31 $$ \theta _{kr}^{(1)} = ( 2r + 1 )\left [ \sum _{j = 0}^{n - k} ( - 1)^{j}(k + j) \begin{pmatrix} n - k \\ j \end{pmatrix} \begin{pmatrix} n + k + j + 1 \\ n - k \end{pmatrix}\sum_{l = 0}^{n - r} \frac{( - 1)^{l}\binom {n - r }{l} \binom {n + r + l + 1 }{n - r} }{k + j + r + l} \right ]. $$

Therefore, for the vector $\Phi (t) $ defined by (13), we get

32 $$ \frac{d\Phi (t)}{dt} = D_{t}\Phi (t) = D^{(1)}\Phi (t), $$

where $D^{(1)} $ is the $(n + 1) \times (n + 1) $ operational matrix of derivative based on the ALPs as follows:

33 $$ D^{(1)} = \bigl[\theta _{k r}^{(1)}\bigr],\quad k,r = 0,1,\ldots,n . $$

## Implementation of an alternative Legendre polynomials method on the novel coronavirus (COVID-19) problem

Firstly, note that the variable of system (1) becomes normalized as follows [4]:

$$ \begin{gathered} s_{p} = \frac{S_{p}}{N_{p}},\qquad e_{p} = \frac{E_{p}}{N_{p}},\qquad i_{p} = \frac{I_{p}}{N_{p}},\qquad a_{p} = \frac{A_{p}}{N_{p}},\qquad r_{p} = \frac{R_{p}}{N_{p}},\qquad w = \frac{\varepsilon W}{\mu _{p}N_{p}},\\ \mu '_{p} = c\mu _{p},\qquad b_{p} = \beta _{p}N_{p},\qquad b_{W} = \frac{\mu _{p}\beta _{W}N_{p}}{\varepsilon }. \end{gathered} $$

So, the normalized model is changed as follows:

34 $$ \textstyle\begin{cases} \frac{ds_{p}}{dt} = n_{p} - m_{p}s_{p} - b_{p}s_{p} ( i_{p} + ka_{p} ) - b_{W}s_{p}w, \\ \frac{de_{p}}{dt} = b_{p}s_{p} ( i_{p} + ka_{p} ) + b_{W}s_{p}w - ( 1 - \delta _{p} )\omega _{p}e_{p} - \delta _{p}\omega '_{p}e_{p} - m_{p}e_{p}, \\ \frac{di_{p}}{dt} = ( 1 - \delta _{p} )\omega _{p}e_{p} - ( \gamma _{p} + m_{p} )i_{p}, \\ \frac{da_{p}}{dt} = \delta _{p}\omega '_{p}e_{p} - ( \gamma '_{p} + m_{p} )a_{p}, \\ \frac{dr_{p}}{dt} = \gamma _{p}i_{p} + \gamma '_{p}a_{p} - m_{p}r_{p}, \\ \frac{dw}{dt} = \varepsilon ( i_{p} + ca_{p} - w ) \end{cases} $$

with the initial conditions

$$ s_{p} ( 0 ) = s_{0},\qquad e_{p} ( 0 ) = e_{0},\qquad i_{p} ( 0 ) = i_{0},\qquad a_{p} ( 0 ) = a_{0},\qquad r_{p} ( 0 ) = r_{0},\qquad w ( 0 ) = w_{0}. $$

The main objective of this paper is to implement ALPs approach on the system of differential Eqs. (34) with the above initial conditions to find the numerical solution of this system. From Eq. (11), we can approximate our unknown functions as follows:

35 $$ \begin{gathered} s_{p} = C_{1}^{T}\Phi (t),\qquad e_{p} = C_{2}^{T}\Phi (t),\qquad i_{p} = C_{3}^{T}\Phi (t),\\ a_{p} = C_{4}^{T}\Phi (t),\qquad r_{p} = C_{5}^{T}\Phi (t),\qquad w = C_{6}^{T} \Phi (t), \end{gathered} $$

where coefficient vectors $C_{i}:i = 1,\ldots,6$ that were defined in Eq. (29) are as follows:

36 $$ \begin{gathered} C_{1} = [c_{0}, \ldots,c_{n}]^{T}, \qquad C_{2} = [c_{n + 1},\ldots,c_{2n + 1}]^{T},\qquad C_{3} = [c_{2n + 2},\ldots,c_{3n + 2}]^{T}, \\ C_{4} = [c_{3n + 3},\ldots,c_{4n + 3}]^{T}, \qquad C_{5} = [c_{4n + 4},\ldots,c_{5n + 4}]^{T}, \qquad C_{6} = [c_{5n + 5},\ldots,c_{6n + 5}]^{T}. \end{gathered} $$

By using Eqs. (34) and (32), we have

37 $$ \textstyle\begin{cases} \frac{ds_{p}}{dt} = C_{1}^{T}\Phi '(t) = C_{1}^{T}D^{(1)}\Phi (t) ,\\ \frac{de_{p}}{dt} = C_{2}^{T}\Phi '(t) = C_{2}^{T}D^{(1)}\Phi (t) ,\\ \frac{di_{p}}{dt} = C_{3}^{T}\Phi '(t) = C_{3}^{T}D^{(1)}\Phi (t) ,\\ \frac{da_{p}}{dt} = C_{4}^{T}\Phi '(t) = C_{4}^{T}D^{(1)}\Phi (t) ,\\ \frac{dr_{p}}{dt} = C_{5}^{T}\Phi '(t) = C_{5}^{T}D^{(1)}\Phi (t) ,\\ \frac{dw}{dt} = C_{6}^{T}\Phi '(t) = C_{6}^{T}D^{(1)}\Phi (t). \end{cases} $$

By substituting Eqs. (37) and (35) into the system of differential Eqs. (1), we have

38 $$ \textstyle\begin{cases} C_{1}^{T}D^{(1)}\Phi (t) = n_{p} - m_{p}C_{1}^{T}\Phi (t) - b_{p}C_{1}^{T} \otimes C_{3}^{T}\Phi (t)\\ \hphantom{C_{1}^{T}D^{(1)}\Phi (t) =}{} - kb_{p}C_{1}^{T} \otimes C_{4}^{T}\Phi (t) - b_{W}C_{1}^{T} \otimes C_{6}^{T}\Phi (t), \\ C_{2}^{T}D^{(1)}\Phi (t) = b_{p}C_{1}^{T} \otimes C_{3}^{T}\Phi (t) + kb_{p}C_{1}^{T} \otimes C_{4}^{T}\Phi (t) + b_{W}C_{1}^{T} \otimes C_{6}^{T}\Phi (t) \\ \hphantom{C_{2}^{T}D^{(1)}\Phi (t) =}{}- ( 1 - \delta _{p} )\omega _{p}C_{2}^{T}\Phi (t) - \delta _{p}\omega '_{p}C_{2}^{T}\Phi (t) - m_{p}C_{2}^{T}\Phi (t), \\ C_{3}^{T}D^{(1)}\Phi (t) = ( 1 - \delta _{p} )\omega _{p}C_{2}^{T}\Phi (t) - ( \gamma _{p} + m_{p} )C_{3}^{T}\Phi (t), \\ C_{4}^{T}D^{(1)}\Phi (t) = \delta _{p}\omega '_{p}C_{2}^{T}\Phi (t) - ( \gamma '_{p} + m_{p} )C_{4}^{T}\Phi (t), \\ C_{5}^{T}D^{(1)}\Phi (t) = \gamma _{p}C_{3}^{T}\Phi (t) + \gamma '_{p}C_{4}^{T}\Phi (t) - m_{p}C_{5}^{T}\Phi (t), \\ C_{6}^{T}D^{(1)}\Phi (t) = \varepsilon ( C_{3}^{T}\Phi (t) + cC_{4}^{T}\Phi (t) - C_{6}^{T}\Phi (t) ). \end{cases} $$

Also, by considering the initial conditions for main problem (1) and Eq. (35), we have

39 $$ \begin{gathered} C_{1}^{T}\Phi (0) = s_{0},\qquad C_{2}^{T} \Phi (0) = e_{0},\qquad C_{3}^{T}\Phi (0) = i_{0},\\ C_{4}^{T}\Phi (0) = a_{0},\qquad C_{5}^{T}\Phi (0) = r_{0},\qquad C_{6}^{T} \Phi (0) = w_{0}. \end{gathered} $$

Equation (39) gives six linear equations.

Since the total unknowns for vectors $C_{i}:i = 1,\ldots,6$ are ($6n + 5$), we collocate Eq. (38) in the set of ($6n - 1$) nodal points $t_{l}$ of the Guass–Chelyshkov [10] as follows:

40 $$ Q_{n} = \bigl\{ t_{l} | P_{n + 1,0}(t_{l}) = 0, l = 0,1,\ldots,n \bigr\} . $$

Now, by replacing the nodes $t_{l}$ in Eq. (38),

41 $$ \textstyle\begin{cases} C_{1}^{T}D^{(1)}\Phi (t_{i}) = n_{p} - m_{p}C_{1}^{T}\Phi (t_{i}) - b_{p}C_{1}^{T} \otimes C_{3}^{T}\Phi (t_{i}) \\ \hphantom{ C_{1}^{T}D^{(1)}\Phi (t_{i}) =}{}- kb_{p}C_{1}^{T} \otimes C_{4}^{T\Phi (t_{i})} - b_{W}C_{1}^{T} \otimes C_{6}^{T}\Phi (t_{i}), \\ C_{2}^{T}D^{(1)}\Phi (t_{i}) = b_{p}C_{1}^{T} \otimes C_{3}^{T}\Phi (t_{i}) + kb_{p}C_{1}^{T} \otimes C_{4}^{T}\Phi (t_{i} + b_{W}C_{1}^{T} \otimes C_{6}^{T}\Phi (t_{i})\\ \hphantom{C_{2}^{T}D^{(1)}\Phi (t_{i}) =}{} - ( 1 - \delta _{p} )\omega _{p}C_{2}^{T}\Phi (t_{i}) - \delta _{p}\omega '_{p}C_{2}^{T}\Phi (t_{i}) - m_{p}C_{2}^{T}\Phi (t_{i}), \\ C_{3}^{T}D^{(1)}\Phi (t_{i}) = ( 1 - \delta _{p} )\omega _{p}C_{2}^{T}\Phi (t_{i}) - ( \gamma _{p} + m_{p} )C_{3}^{T}\Phi (t_{i}), \\ C_{4}^{T}D^{(1)}\Phi (t_{i}) = \delta _{p}\omega '_{p}C_{2}^{T}\Phi (t_{i}) - ( \gamma '_{p} + m_{p} )C_{4}^{T}\Phi (t_{i}), \\ C_{5}^{T}D^{(1)}\Phi (t_{i}) = \gamma _{p}C_{3}^{T}\Phi (t_{i}) + \gamma '_{p}C_{4}^{T}\Phi (t_{i}) - m_{p}C_{5}^{T}\Phi (t_{i}), \\ C_{6}^{T}D^{(1)}\Phi (t_{i}) = \varepsilon ( C_{3}^{T}\Phi (t_{i}) + cC_{4}^{T}\Phi (t_{i}) - C_{6}^{T}\Phi (t_{i}) ) \end{cases} $$

for $i = 0,\ldots,6n - 1$, we can solve this system of 6*n* equations that resulted from Eqs. (39) and (41) by using Newton’s iteration scheme [12–16] for calculating the unknown vectors $C_{i}:i = 1,\ldots,6$.

For existence and stability of the proposed method with ALPs, we can refer to paper [9].

In our implementation, the calculations are done in *Mathematica 11* software, on a personal computer with Core-i5 processor, 2.67 GHZ frequency, and 4 GB memory.

## Numerical example

In this section a test problem of the coronavirus model is solved by our proposed method.

The values of the initial conditions and parameters are given as [4]:

$$ \begin{gathered} N_{p} = 1\mbox{,}000\mbox{,}000 \mbox{,}000,\qquad \delta _{p} = k = \beta _{p} = \beta _{w} = \mu _{p} = \gamma '_{p} = c = 0.5,\qquad {n}_{p} = m_{p} = 0.0018, \\ \varepsilon = 0.1,\qquad w_{p} = w'_{p} = 0.1923,\qquad \gamma _{p} = 0.1724. \end{gathered} $$

Also, we get the initial values of unknown parameters as follows:

$$ s_{p} ( 0 ) = 2,\qquad e_{p} ( 0 ) = 4, \qquad i_{p} ( 0 ) = 3,\qquad a_{p} ( 0 ) = 4,\qquad r_{p} ( 0 ) = 2,\qquad w ( 0 ) = 3.5. $$

**Figure 2:**
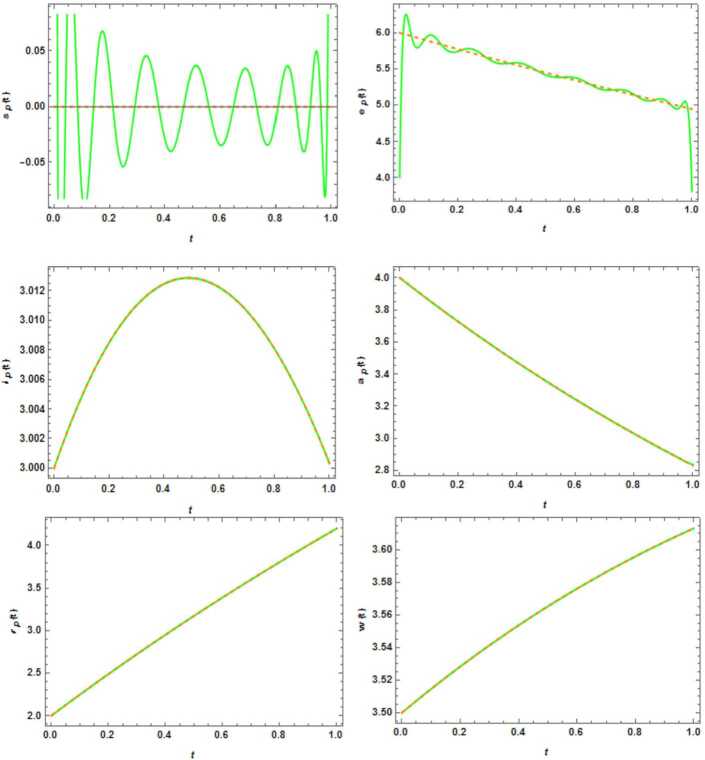
Comparison of the computed and RK4 solutions for functions $s_{p}(t)$, $e_{p}(t)$, $i_{p}(t)$, $a_{p}(t)$, $r_{p}(t)$, $w(t) $ with $n = 16$ on the interval $[0,1]$, where the dash shows the RK4 solution and the line shows the ALPs solution

We solve this problem by $n = 16$ in ALPs. The results of proposed method are compared with the results of Runge–Kutta method. Figure 2 and Tables 2–7 show the comparison between them.

**Table 2:** Numerical comparison for $s_{p}(t)$ with $n = 16$ on the interval $[0,1]$

*t*	RK4	Present method
0.0	2.000000000	2.00000000
0.2	1.59905E − 13	0.03002862
0.4	1.59871E − 13	−0.02923894
0.6	1.59898E − 13	−0.03456393
0.8	1.59982E − 13	−0.01139075
1	1.60124E − 13	1.12482514

**Table 3:** Numerical comparison for $e_{p}(t)$ with $n = 16$ on the interval $[0,1]$

*t*	RK4	Present method
0.0	4	4
0.2	5.77189614477	5.74277771538
0.4	5.55247760052	5.58266126424
0.6	5.34141367556	5.37685723359
0.8	5.13838625205	5.15034353745
1	4.94308933537	3.80689507404

**Table 4:** Numerical comparison for $i_{p}(t)$ with $n = 16$ on the interval $[0,1]$

*t*	RK4	Present method
0.0	3	3
0.2	3.00849107875	3.00849108363
0.4	3.01246360734	3.01246360563
0.6	3.01223326389	3.01223326126
0.8	3.00809881420	3.00809880689
1	3.00034288194	3.00034287358

**Table 5:** Numerical comparison for $a_{p}(t)$ with $n = 16$ on the interval $[0,1]$

*t*	RK4	Present method
0.0	4	4
0.2	3.72569099766	3.725275447302
0.4	3.47348293584	3.473083082484
0.6	3.24142145708	3.241082166196
0.8	3.02773254616	3.027574786909
1	2.83080613397	2.836696723317

**Table 6:** Numerical comparison for $r_{p}(t)$ with $n = 16$ on the interval $[0,1]$

*t*	RK4	Present method
0.0	2	2
0.2	2.48888268589	2.48882899953
0.4	2.95149948421	2.95140029238
0.6	3.38981976531	3.38968102039
0.8	3.80563689580	3.80546221628
1	4.20058431510	4.20029857270

**Table 7:** Numerical comparison for $w(t)$ with $n = 16$ on the interval $[0,1]$

*t*	RK4	Present method
0.0	3.5	3.5
0.2	3.52841252047	3.52840049359
0.4	3.55378078473	3.55375851466
0.6	3.57628769500	3.57625646333
0.8	3.59610020735	3.59606081175
1	3.61337096445	3.61330663649

## Conclusion

The World Health Organization declared the coronavirus (COVID-19) a pandemic on March 11, 2020. This virus spread quickly in more than 200 countries, and up to now more than 28 million around the world have been infected. This paper aims to solve the mathematical model of coronavirus that can show the transmissibility of this virus that is vital to measure the progression of the disease and to get a better cure. By use of alternative Legendre polynomials and their operational matrix of derivative, we convert the system of coronavirus model to an algebraic model. We compare the results of the present method with those of the Runge–Kutta method, which confirmed the reliability of the proposed method results.
